# Innovative Virtual Role Play Simulations for Managing Substance Use Conversations: Pilot Study Results and Relevance During and After COVID-19

**DOI:** 10.2196/27164

**Published:** 2021-04-29

**Authors:** Glenn Albright, Nikita Khalid, Kristen Shockley, Kelsey Robinson, Kevin Hughes, Bethany Pace-Danley

**Affiliations:** 1 Baruch College Department of Psychology City University of New York New York, NY United States; 2 The Graduate Center City University of New York New York, NY United States; 3 Department of Psychology University of Georgia Athens, GA United States; 4 Peer Assistance Services, Inc Denver, CO United States

**Keywords:** simulations, behavior change, motivational interviewing, virtual humans, role play, substance use, prevention, alcohol, public awareness, innovation, interview, COVID-19, pilot study, simulation, communication, problem solving

## Abstract

**Background:**

Substance use places a substantial burden on our communities, both economically and socially. In light of COVID-19, it is predicted that as many as 75,000 more people will die from alcohol and other substance use and suicide as a result of isolation, new mental health concerns, and various other stressors related to the pandemic. Public awareness campaigns that aim to destigmatize substance use and help individuals have meaningful conversations with friends, coworkers, or family members to address substance use concerns are a timely and cost-effective means of augmenting existing behavioral health efforts related to substance use. These types of interventions can supplement the work being done by existing public health initiatives.

**Objective:**

This pilot study examines the impact of the *One Degree: Shift the Influence* role play simulation, designed to teach family, friends, and coworkers to effectively manage problem-solving conversations with individuals that they are concerned about regarding substance use.

**Methods:**

Participants recruited for this mixed methods study completed a presurvey, the simulation, and a postsurvey, and were sent a 6-week follow-up survey. The simulation involves practicing a role play conversation with a virtual human coded with emotions, a memory, and a personality. A virtual coach provides feedback in using evidence-based communication strategies such as motivational interviewing.

**Results:**

A matched sample analysis of variance revealed significant increases at follow-up in composite attitudinal constructs of preparedness (*P*<.001) and self-efficacy (*P*=.01), including starting a conversation with someone regarding substance use, avoiding upsetting someone while bringing up concerns, focusing on observable facts, and problem solving. Qualitative data provided further evidence of the simulation’s positive impact on the ability to have meaningful conversations about substance use.

**Conclusions:**

This study provides preliminary evidence that conversation-based simulations like *One Degree: Shift the Influence* that use role play practice can teach individuals to use evidence-based communication strategies and can cost-effectively reach geographically dispersed populations to support public health initiatives for primary prevention.

## Introduction

### Introduction to Substance Use

#### Prevalence and Outcomes of Substance Use in the United States

According to the Substance Abuse and Mental Health Services Administration National Survey on Drug Use and Health (NSDUH), 20.3 million Americans 12 years and older had been diagnosed with a substance use disorder in 2018 [1]. Alcohol is the most commonly used substance in the United States with 139.8 million people 12 years and older reporting having drank alcohol in the past month at the time of the NSDUH [1]. Although most people who use alcohol do not have a substance use disorder, many still drink at levels that can be hazardous to their health. For example, 67.1 million Americans reported binge drinking in the past month, and another 16.6 million reported heavy drinking in the past month [1]. Aside from short-term risks such as accidents, injuries, and alcohol poisoning, long-term excessive alcohol use can contribute to cancers, high blood pressure, and heart disease among other illnesses, and can exacerbate existing health conditions [2]. Further, alcohol is the third leading cause of preventable death in the United States. From 2011 to 2015, alcohol contributed to an average of 93,000 deaths annually, accounting for a total of 2.7 million years of potential life lost [2].

The impact of substance use on communities can be devastating. In addition to injury, illness, and social consequences, there are also exponential economic costs. In 2010, the impact of excessive alcohol use alone was US $249.0 billion nationally with a median cost of US $3.5 billion per state [3]. These costs include losses in workplace productivity (72%); health care expenses (11%); and additional costs for motor vehicle accidents, property damage, and criminal justice expenses [2,4]. Despite this, the US $35.6 billion National Drug Control Strategy budget allocates 45.1% to treatment yet only 17% (US $2.1 billion) goes toward prevention, a number that has steadily decreased every year since 2018 [5]. Prevention programs are essential for lessening the public health burden of alcohol and other substance use in our communities, and there is a need for implementation of effective initiatives that mitigate the physical, social, mental, and economic consequences of substance use.

#### Prevalence and Outcomes of Substance Use in Colorado

The state of Colorado, where this pilot study was conducted, has recognized and is responding to the prevalence of substance use and substance use disorders within its communities as evidenced by funding new and innovative approaches like the one outlined in this study. In Colorado alone, 16.3% of the population 12 years and older have been diagnosed with an alcohol or substance use disorder, which is approximately 927,000 people [6]. Approximately 1 million Colorado adults, 27% of the state’s adult population, indicate that they themselves, or someone that they know, has been addicted to alcohol or another substance in their lifetime [7]. Further, one in five Colorado adults report binge drinking, and excessive drinking costs the state roughly US $5 billion each year [3,8]. In addition, as of 2019, Colorado had the fifth highest number of alcohol-related deaths compared to other US states, averaging 5 deaths per day due to excessive drinking [9,10].

Colorado also has progressive legislation surrounding cannabis use, which adds an additional layer to the social and legal impact of substance use among residents. As the culture of cannabis use shifts, it is increasingly subjective and difficult to recognize when substance use progresses into a substance use disorder. According to a 2018 study, 17.5% of Colorado adults were current cannabis users and of those who reported using cannabis in the past 30 days, 51.5% reported that they used it either daily or near daily [9].

#### Substance Use and COVID-19

The collective impact of COVID-19 has resulted in substantial stress associated with unemployment, mandated social isolation, grief and loss, and the many other collateral consequences that increase the susceptibility to substance use, addiction, and relapse [11,12]. June 2020 research found that 13.3% of US adults reported having started or increased substance use as a direct result of coping with stress, new or worsened depression and anxiety, or other emotions related to COVID-19 [13]. This research noted that younger adults, racial and ethnic minorities, essential workers, and unpaid adult caregivers reported experiencing negative mental health outcomes, increases in substance use and increased suicidal ideation at a disproportionally high rate compared to other groups of people [13]. Studies also show that, post disaster, people can exhibit psychological distress or trauma, thus are more likely to initiate or increase alcohol or prescription and illicit drug use [14]. It is predicted that there will be as many as 75,000 more preventable deaths from alcohol and other substance use, and suicide in the coming years due to isolation, mental health concerns, and various other stressors related to the pandemic [15,16].

#### Substance Use and Stigma

Stigmatization of substance use and mental health is perpetuated by a number of different factors, including blame, stereotypes, a lack of knowledge around mental health and substance use disorders, a lack of personal contact with people who have experienced substance use, and negative media portrayals [17]. According to 2018 NSDUH data, nearly 15% of individuals who indicated that they needed substance use treatment in the past year, but did not receive it, reported that they avoided seeking treatment because they “felt that getting treatment would cause their neighbors or community to have a negative opinion of them” [1]. Similarly, the Colorado Health Institute reported that over 70% of respondents who needed but did not receive substance use treatment in 2019 indicated that the main reasons for not seeking help for substance use were that they were afraid someone would find out that they had a problem or that they did not feel comfortable talking about personal issues [7].

An additional challenge in Colorado and other states with legalized cannabis is understanding the cultural aspect of recreational drug use in the United States, as it can impact stigma regarding help seeking for those with a more serious disorder. It can also lead to misunderstandings regarding the health-related risks of recreational drug use. Similarly, normalization and general acceptability of alcohol use across the United States combined with its place in social and celebratory environments may lead to risky and excessive alcohol use that is not properly addressed in many health care settings. Stigmatization and normalization of certain substances can negatively impact opportunities for conversations around substance use and may stop people from seeking treatment.

### The One Degree Simulation

#### Background

The *One Degree: Shift the Influence* is a Colorado public awareness campaign consisting of virtual human role play simulations in which individuals can practice having conversations with loved ones about substance use. The main goals of the public awareness campaign are to decrease stigma around substance use and to inspire others to seek help as needed through meaningful and effective conversation. The simulation was developed by Kognito in collaboration with Peer Assistance Services, Inc and with input from nationally recognized subject matter experts in the fields of mental health, nursing, public health, social work, and health education. Peer Assistance Services is a Colorado-based nonprofit agency, leading with prevention and intervention for substance use and mental health concerns.

**Figure 1:**
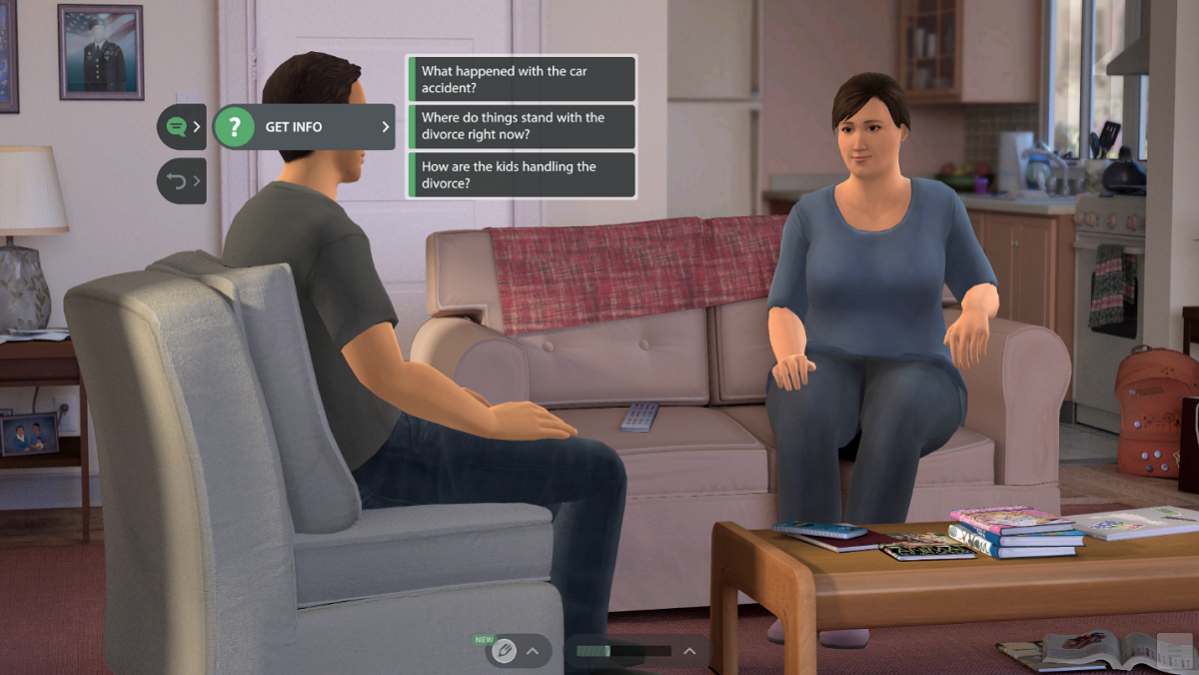
Example of dialogue options to build rapport between characters Phil and Donna.

The *One Degree: Shift the Influence* simulation is built around a series of mini conversations where users interact with intelligent, fully animated, and emotionally responsive virtual humans experiencing the negative effects of alcohol or cannabis use. Possessing their own personalities and memories, these virtual humans adapt their verbal and nonverbal responses to the conversation tactics or dialogue options that participants select throughout the role play. The dialogue options represent a variety of effective, neutral, and ineffective tactics in managing a conversation and are controlled by a set of mathematical behavioral models and algorithms specifically designed to simulate real interactions. These algorithms permit the learner to continually experience the consequences of their dialogue selections within the role play to develop skills and knowledge. In some cases, a tactic that is ineffective at one point in the conversation may be effective elsewhere. Once learners choose a dialogue option, they see their virtual human *perform* the dialogue and then observe the response of the virtual human. A new set of dialogue options then appears based on which tactic was selected (an example of which can been seen in Figure 1). If the participant selects choices that include being critical, judgmental, or labeling, the virtual human will react negatively to the tactic, thus providing immediate feedback to the learner. Throughout the simulation, participants are able to occasionally view the virtual human’s private thoughts, which are designed to provide the learner with greater insight and understanding, thus fostering empathic communication skills. In addition, a virtual coach occasionally provides positive feedback for selecting effective dialogue tactics and corrective feedback for selecting ineffective ones. The role play is complete once the participant successfully uses evidence-based conversation tactics such as motivational interviewing (MI) that build the virtual human’s trust, resulting in opportunities to discuss substance use concerns in a helpful way.

#### Simulation Platform and Efficacy Research

The simulation is based on a digital conversation platform that includes an innovative group of development, delivery, an application programming interface, data collection, and analytic technologies that integrate evidence-based communication strategies that include elements of mindfulness, emotional regulation, empathy, adult learning theory, and the four core MI skills. These MI skills include (1) asking open-ended questions, (2) providing affirmation, (3) reflective or active listening (listening closely and periodically confirming comprehension), and (4) summarizing what was said. MI, originally developed by Miller [18,19] to address problem drinking, has been shown in numerous meta-analytic studies to be an effective modality for bringing about behavioral change in various clinical contexts [20-28]. Traditionally, MI is most effective when preceded by a “teachable moment ” [21]. In a clinical setting, this may be a visit to an emergency department following an incident directly related to substance use or in a primary care setting when a patient presents with concerns such as worsened depression or anxiety, which can be directly attributed to substance use. In the *One Degree: Shift the Influence* simulations, conversations also follow teachable moments, which provides an opportunity for the subject of substance use to be approached naturally. With Donna, one of the two character options, the teachable moment comes when a friend of hers expresses concern to her cousin, Phil, about the amount of alcohol that she has been drinking at recent happy hours. For the conversation with Jordan, the other character option, the teachable moment involves repeated occurrences of Jordan coming in late to work or missing shifts entirely due to excessive partying the night before.

Numerous studies have examined the efficacy of using virtual human role play simulations, similar to that of *One Degree: Shift the Influence*, to teach MI skills. These studies have found that virtual role play practices can provide an efficacious means of delivering screening and brief intervention training for health care providers [29-34], and have proven to successfully change attitudinal constructs and behaviors in K12 and higher education settings related to identifying; talking to; and, if necessary, referring students in psychological distress. [29,35-39]. Virtual role play simulations that integrated MI techniques were equally effective across multiple races and ethnicities including people who were Black, Hispanic, Latinx, White, Asian, and American Indian or Alaska Native [40,41].

#### Advantages of Role Play Simulations

The success of virtual role plays in having a positive impact on attitudinal constructs that predict behavior such as increasing learner preparedness and self-efficacy is partly due to the ability to create realistic and contextually appropriate role plays in an environment that is risk-free and confidential. The virtual learning promotes skill building at one’s own pace without concern for making mistakes in a public forum, such as live instructor driven role plays with other learners present. Learners are not at the center of attention, which helps to avoid social evaluative threat and anxiety; thus, they are less likely to feel judged or embarrassed and more likely to be themselves and reveal information [42,43].

Finally, due to the algorithms specifically designed to simulate real interactions, trainer bias and possible fatigue is eliminated, and content can be presented with high fidelity, optimizing the learning experience [29]. This means that the virtual humans will consistently respond verbally and nonverbally in the most efficacious way to promote skill development and drive behavior change.

#### Simulation Story Lines Overview

The story lines developed for the simulation were the result of an iterative process between subject matter experts, instructional designers, and Peer Assistance Services of Colorado. The criteria established was that they would have to appeal to the broadest group of people; be a family member, friend, or coworker; and vary in ages.

The first role play conversation is with Donna, a single mom who is going through a difficult divorce. She has always been outgoing, extroverted, and successful at work, but lately, she has been drinking more to cope with the stress. Now, Donna is having two or three drinks each night, even on nights when she is not with her friends. She has also started relying on alcohol to help her get to sleep. Donna’s friends and family have noticed that she has been acting differently than her usual self. In the simulation, the learner plays the role of Phil, a relative who is concerned about Donna’s increased drinking as a coping mechanism for her stressful life. The learner will practice how to bring up their concerns without upsetting Donna and help her brainstorm alternative ways to cope with stress. The goal when creating Donna was to characterize unhealthy alcohol use that was not at the level of alcohol use disorder and to demonstrate how a person may turn to alcohol to cope with everyday stressors. Donna represents a middle-aged woman facing a significant common life stressor, divorce, while also balancing the challenges of parenting and supporting her children. Donna’s story presents an opportunity to highlight parenting and role-modeling appropriate adult use of alcohol use as a possible motivating factor for a person to change alcohol use. Coping with stress and symptoms of depression are common reasons that lead people to drink too much and often the person is unaware that, over time, alcohol can actually make stress and depression worse. Insomnia is also a common complaint, and alcohol plays an important role in the quality of sleep a person is getting. Lack of sleep or poor quality sleep can make stress and depression worse because healthy sleep is so critical for overall emotional well-being. Both of these common health concerns are demonstrated in Donna’s story. Her story allows individuals who do not have a background in health care to understand some of the most common reasons that people may begin using substances and speaks to some common health outcomes of excessive use.

The second conversation is with Jordan, a young adult who has been thinking about going to college. He’s been saving money by working in restaurants while living with his parents. Jordan used to enjoy being outdoors and camping on the weekends, but recently, he has been spending most of his time and savings on partying with his friends, smoking cannabis, and drinking. He is routinely intoxicated, and it has been affecting his work performance and relationships, including with his boss. In this simulation, the learner will play the role of Phil, a coworker who is concerned about Jordan. The learner will practice how to bring up their concerns without upsetting Jordan and brainstorm ways to balance his partying with his goal of saving money and going to college. The primary goal with this character was to characterize how cannabis use can affect aspects of life other than health, especially because sometimes health issues take longer to develop. Young adult males are more likely to use cannabis frequently. In addition, especially since the legalization of cannabis in Colorado, its use has continued to increase among adults at the same time that the public’s perception of harm is decreasing. Because of this, recreational cannabis use may be a common concern seen among friends and family members of individuals using this simulation. Jordan is a young adult using cannabis in ways that may seem ordinary and unlikely to cause major problems but that can actually begin to interfere with his motivation and capacity to reach his goals of saving money and returning to school. Cannabis and alcohol use can become financially costly, and some people choose to change cannabis, alcohol, or tobacco use to use the money for other things that matter to them. The effects of cannabis on motivation and ability to reach personal goals can be fairly subtle and unrecognized by the individual, and a compassionate friend can sometimes help a person identify that this is happening, and the person will make a change before the pattern of use becomes far more difficult to change.

In both scenarios, the role play begins with a didactic introduction from Phil. Given that these role play scenarios are directed at the public rather than at behavioral health professionals, he provides some tips to be mindful of while completing the simulations. He explains the character’s background and then provides an overview of the order that the conversation should be approached: bring up concerns without upsetting the other person, discuss the other person’s stressors or goals, and help the other person problem solve if they are open to it. Phil also cautions that how well this conversation goes will depend on how the other person is feeling but explains that there are strategies that can be used to improve the odds that this will go well, which will be learned and practiced through this simulation. This gives the individual that is interacting with the simulation realistic expectations and an understanding that not all conversations will go as planned. Although not explicitly described as motivational interviewing, the final tips that Phil gives are clearly rooted in the principals of MI: stick to the facts, show you understand, and ask questions.

Throughout the simulations, if the learner selects conversation tactics that cause the virtual human to feel judged, offended, or otherwise have a negative emotional reaction, the learner will be prompted to undo their last action. The learner is then required to pick a different tactic based on the coach’s recommendation. The purpose of the coach is to provide a baseline understanding of how to use MI techniques to elicit behavior change; therefore, coaching suggestions are rooted in best practices for MI. The coach does not introduce any additional information related to risk factors or facts about substance use. However, if the learner seeks information or guidance on additional topics related to substance use, they can easily locate that information at the end of the simulation. Upon completion, participants view a dashboard that provides an overview of their performance, including feedback on how well they met different goals throughout the conversation. This dashboard also links back to the Shift the Influence website, which houses a number of resources such as fact sheets, crisis lines, treatment locators, and other statewide public awareness campaigns.

### Objective of This Study

The aim of this study is to examine the impact of the *One Degree: Shift the Influence* simulation on participant ability to engage in and effectively manage conversations with individuals they have concerns about due to their substance use. We hypothesize that the simulation will increase participant preparedness, likelihood or behavioral intent, and self-efficacy to initiate a conversation, avoid upsetting someone when bringing up concerns, focus on observable facts, and problem solve. An additional aim is to observe changes in both personal and public stigma regarding substance use as a result of using this app. By addressing these key areas, we aim to provide preliminary evidence that community members can play an active role in ameliorating a major public health problem by learning from this new and innovative teaching tool, and normalizing having conversations about substance use in their daily life.

## Methods

### Recruitment

A total of 80 participants were recruited for this mixed methods pilot study by responding to an ad in regional press publications covering four counties in the state where Colorado’s *One Degree: Shift the Influence* campaign focused their marketing efforts. The ad stipulated that we were seeking people to participate in a study to evaluate the effectiveness of a game-based virtual human (avatar) role play simulation that teaches individuals how to manage a conversation with someone that they are concerned about regarding their alcohol or other substance use. Participants were informed by email that they would need to take a short presurvey (baseline), then complete a 30-minute online role play simulation, followed by an immediate postsurvey and a 6-week follow-up survey. Participants received a US $30 gift card upon completing the simulation with the associated pre- and postsurveys, and a US $20 gift card after completing the follow-up survey. The entire study required approximately 45-60 nonconsecutive minutes.

To qualify, participants must have been 18 years or older and had access to a computer with audio and internet capabilities. Upon agreeing to participate, participants received a link to the study that led them to the informed consent page and the Survey Monkey hosted presurvey. After completing the presurvey, they selected and completed one of two role play simulations followed by a postsimulation survey. They were emailed a follow-up survey 6 weeks later. The Baruch College Human Research Protection Program/Institutional Review Board and Peer Assistance Services, Inc, a Colorado-based nonprofit agency, determined that no ethics approval was required for this study.

### Statistical Analysis

#### Quantitative Measures

Kirkpatrick’s [44,45] training evaluation model was used in assessing the impact of the *One Degree: Shift the Influence* simulation. This model evaluates four levels: reaction, learning, behavior, and results. Level one, reaction, is the level of user satisfaction with the training. Level two, learning, is the impact on attitudes, knowledge, or skills. Level three, behavior, represents the change in behavior. Level four, results, are final outcomes such as overall long-term benefits that could include a shift in culture or return on investment. The fourth level was not assessed for it was not within the scope of this study.

Level one assessment questions were asked in the postsurvey immediately after participants completed the simulation. They included:

- Overall, how would you rate the course (five-point Likert scale from “very poor,” 1, to “excellent,” 5)?
- Would you recommend this simulation to a friend or colleague (“yes” or “no”)?
- Is the simulation based on scenarios that are relevant to you (“yes” or “no”)?

Level two survey questions were asked in the pre-, post- and follow-up surveys and included ten items that assessed three attitudinal constructs including participant (1) preparedness, (2) likelihood (or behavioral intent), and (3) self-efficacy. Specifically, these items were drawn from the validated Gatekeeper Behavior Scale [46] and modified for the purpose of this study. This was accomplished by drawing from social cognitive theory [47], Bandura’s [48] integrative framework of personal efficacy to assess preparedness and self-efficacy, and the theory of reasoned action [49,50] to assess behavioral intention or likelihood. All three theories act as a direct precedent of behavior; thus, the three attitudinal construct measures include:

1. Preparedness to engage in helping behaviors related to substance use was measured with four items, which were averaged to create a composite score (Cronbach alpha .82). Responses were set on a five-point Likert scale that ranged from (1) “very low” to (5) “very high.” The common stem for all items was “Please rate your preparedness to” start a conversation about substance use with someone you are concerned about, avoid upsetting someone while bringing up concerns about their substance use, focus on observable facts while bringing up concerns about their substance use, and problem solve with someone to help them address their substance use.
2. Likelihood was measured with two items, which were averaged to create a composite score (Cronbach alpha .72). Responses were set on a five-point Likert scale from (1) “very unlikely” to (5) “very likely.” The common stem for all items was “How likely are you to” start a conversation about substance use with someone you are concerned about and problem solve with someone to help them address their substance use.
3. Self-efficacy was measured with four items, which were averaged to create a composite score (Cronbach alpha .83). Responses were set on a five-point Likert scale from (1) “strongly disagree” to (5) “strongly agree.” The common stem for all items was “Please indicate how much you agree or disagree with the following statements”: “I feel confident in my ability to” start a conversation about substance use with someone you are concerned about, avoid upsetting someone while bringing up concerns about their substance use, focus on observable facts while bringing up concerns about their substance use, and problem solve with someone to help them address their substance use.

Level two survey questions also included measures of social and subjective norms, which are components of the theory of planned behavior and are correlated with helping and help-seeking behaviors [50]. For example, one’s public stigma regarding whether most people approve or disapprove of a behavior influences their decisions to engage in behaviors because it reflects on how aligned those behaviors are with their sense of self and with the community. Personal and public stigma were comprised of two items, for which Cronbach alpha was not calculated as each stigma item was assessed separately (eg, public vs private or personal stigma). Responses were set on a five-point Likert scale from (1) “strongly disagree” to (5) “strongly agree.” The common stem for all items was “Please indicate how much you agree/disagree with the following statements”:

- Most people think less of a person who has been in treatment for substance use (public stigma).
- I think less of a person who has been in treatment for substance use (personal stigma).

Level three survey questions were asked in the pre- and follow-up surveys. Self-reported behavioral measures included “in the past 6 weeks, approximately how many times have you: started a conversation about substance use with someone you are concerned about? Problem solved with someone to help them address their substance use? Consulted with a health professional about substance use?”

The quantitative statistical analysis includes descriptive data for level one, a repeated measures analysis of variance (ANOVA) for level two as there were three measurement points to compare (pre, post, and follow-up), and a paired sample *t* test for level three as there were only two comparison points (pre to follow up). All analyses were conducted using SPSS version 26 (IBM Corp). For the repeated measures ANOVA, separate analyses were run for each of the outcome variables (ie, each preparedness item, composite preparedness, each likelihood item, composite likelihood, each self-efficacy item, composite self-efficacy, and both stigma items). In cases where the overall F value was significant, post hoc tests were conducted with a Bonferroni adjustment to correct for type I error. The paired samples *t* tests were also conducted separately for each of the three behavioral variables.

#### Qualitative Measures

Qualitative measures were asked in the post and follow-up survey and included:

- Now that you have completed the simulation, please describe a situation that you would have managed differently. What happened and what would you have done differently? Please do not include any names of people (asked immediately after training in postsurvey).
- Now that you have completed the simulation, can you recall a situation where you used the skills learned in the simulation? Please describe what happened and be sure not to include any names of people (asked at follow-up survey).

The qualitative analysis involved coding for reoccurring themes using a joint inductive–deductive coding process (see Shockley et al [51] for a similar example). This involved two independent coders where the first coder read through the various questions and identified common themes; the second coder did the same, adding and refining categories where applicable; once a final coding template was established, both coders independently coded the responses into the full set of thematic categories; the head coder reviewed the coding for agreement and resolved any discrepancies through discussion with the other coder; the head coder organized the thematic categories into higher order themes as reported in a later section; and the head coder chose quotes that best represented each theme for further illustration. For all content categories, only those with at least 2 statements fitting into that category were reported. Percentages do not add to 100% because a single statement could fit into multiple categories. Statements have been copied verbatim (typos were not corrected).

## Results

### Descriptive Statistics

There were 80 participants recruited for this study whose average age was 31.01 (SD 10.66) years, with 50% (n=40) female, 45% (n=36) male, 1.3% (n=1) gender nonconforming, and 3.8% (n=3) preferring not to answer. Race/ethnicity and employment status can be seen in Table 1.

After completing the first part of the study (presurvey, the simulation, and the postsurvey), 28 participants dropped out. A chi-square compared the differences between those participants who completed the entire study (N=80) to those who did not complete the follow-up survey (n=28). Participants who completed all three survey time points had a significantly higher presurvey score for preparedness (*P*=.01) and self-efficacy (*P*=.02) compared to those who did not complete the follow-up survey. There were no other significant differences in dependent variables including attitudinal measures, age, gender, ethnicity, simulation rating, and satisfaction measures.

**Table 1:** Participant demographics (N=80).

Demographics			Participants
Age (years), mean (SD)			31.01 (10.66)
**Gender, n (%)**			
	Female	40 (50)	
	Male	36 (45)	
	Gender nonconforming or other gender identity	1 (1.3)	
	Prefer not to answer	3 (3.8)	
**Race or ethnicity, n (%)**			
	White	55 (68.8)	
	Black or African American	5 (6.3)	
	Hispanic or Latinx	11 (13.8)	
	American Indian/Alaska Native	1 (1.3)	
	Asian	5 (6.3)	
	Native Hawaiian/Other Pacific Islander	2 (2.5)	
	Prefer not to answer	7 (8.8)	
**Employment status, n (%)**			
	Full time	41 (51.3)	
	Part time	16 (20)	
	Not working	17 (21.3)	
	Prefer not to answer	6 (7.5)	

### Quantitative Measures

Level one satisfaction findings showed that 100% of all 80 participants rated the simulation either excellent (n=24, 30%), very good (n=41, 51%), or good (n=15, 19%). Additionally, 95% (n=76) stated they would recommend the simulation to a friend, and 84% (n=67) reported that the simulation was based on scenarios that were relevant to them.

Table 2 shows descriptive statistics for individual and composite scores across all three survey time points and shows the results of the repeated measures ANOVA analysis, post hoc tests, and effect size information (partial eta^2^). Similar tables are shown for the likelihood (Table 3) and self-efficacy (Table 4) attitudinal constructs. The results show that preparedness and self-efficacy composite attitudinal measures significantly increased from the presurvey to the follow-up survey after post hoc adjustment. The likelihood construct did not maintain its significance after the post hoc correction.

The stigma findings show a slight nonsignificant decrease in both private and public stigma (see Table 5).

**Table 2:** Preparedness descriptive statistics and repeated measures ANOVA results.

Preparedness^a^			Response, mean (SD)^b^		Repeated measures ANOVA^c^, *F* value		*P* value		Post hoc tests, mean difference							*P* value							Partial eta^2^ for F
									Pre to post		Pre to follow-up		Post to follow-up		Pre to post			Pre to follow-up		Post to follow-up			
**Start a conversation about substance use with someone you are concerned about**					16.09		<.001		0.37		0.64		0.27		.005			<.001		.04		0.24	
	Pre	3.40 (1.05)																					
	Post	3.77 (0.81)																					
	Follow-up	4.04 (0.74)																					
**Avoid upsetting someone while bringing up concerns about their substance use**					13.46		<.001		0.43		0.69		0.27		.02			.001		.09		0.21	
	Pre	3.17 (094)																					
	Post	3.60 (0.89)																					
	Follow-up	3.87 (0.74)																					
**Focus on observable facts while bringing up concerns about their substance use**					5.62		.005		0.27		0.44		0.17		.23			.006		.39		0.10	
	Pre	3.62 (0.95)																					
	Post	3.88 (0.83)																					
	Follow-up	4.06 (0.70)																					
**Problem solve with someone to help them address their substance use**					9.33		<.001		0.46		0.58		0.12		.008			.004		.78		0.16	
	Pre	3.52 (1.04)																					
	Post	3.98 (0.70)																					
	Follow-up	4.10 (0.82)																					
**Composite preparedness**					17.71		<.001		0.38		0.59		0.21		.004			<.001		.03		0.26	
	Pre	3.42 (0.85)																					
	Post	3.81 (0.69)																					
	Follow-up	4.01 (0.58)																					

^a^Each item begins with “How would you rate your preparedness to...”

^b^n=52 for all time points. All preparedness items are the same across all survey time points.

^c^ANOVA: analysis of variance.

**Table 3:** Likelihood descriptive statistics and repeated measures ANOVA results.

Likelihood^a^			Response, mean (SD)^b^		Repeated measures ANOVA^c^, F value		*P* value		Post hoc tests, mean difference							*P* value							Partial eta^2^ for F
									Pre to post		Pre to follow-up		Post to follow-up		Pre to post			Pre to follow-up		Post to follow-up			
**Start a conversation about substance use with someone you are concerned about?**					4.55		.01		0.27		0.33		0.06		.05			.06		>.99		0.10	
	Pre	3.73 (0.97)																					
	Post	4.00 (0.71)																					
	Follow-up	4.06 (0.64)																					
**Problem solve with someone to help them address their substance use?**					1.03		.36		N/A^d^		N/A		N/A		N/A			N/A		N/A		0.02	
	Pre	4.12 (0.65)																					
	Post	4.15 (0.57)																					
	Follow-up	4.25 (0.59)																					
**Composite** **likelihood**					3.15		.047		0.15		0.23		0.08		.25			.14		.97		0.06	
	Pre	3.92 (0.73)																					
	Post	4.07 (0.55)																					
	Follow-up	4.15 (0.54)																					

^a^Each item begins with “How likely are you to...”

^b^n=52 for all time points. All likelihood items are the same across all survey time points.

^c^ANOVA: analysis of variance.

^d^N/A: not applicable.

**Table 4:** Self-efficacy descriptive statistics and repeated measures ANOVA results.

Self-efficacy^a^			Response, means (SD)^b^		Repeated measures ANOVA^c^, F value		*P* value		Post hoc tests, mean difference						*P* value						Partial eta^2^ for F
									Pre to post		Pre to follow-up		Post to follow-up		Pre to post		Pre to follow-up		Post to follow-up		
**Start a conversation about substance use with someone you are concerned about**					11.23		<.001		0.54		0.50		0.04		<.001		.007		>.99		0.18
	Pre	3.52 (1.09)																			
	Post	4.06 (0.57)																			
	Follow-up	4.02 (0.75)																			
**Avoid upsetting someone while bringing up concerns about their substance use**					8.46		<.001		0.46		0.54		0.08		.01		.005		>.99		0.14
	Pre	3.29 (0.98)																			
	Post	3.75 (0.76)																			
	Follow-up	3.83 (0.76)																			
**Focus on observable facts while bringing up concerns about their substance use**					1.87		.16		N/A^d^		N/A		N/A		N/A		N/A		N/A		0.035
	Pre	3.92 (0.79)																			
	Post	4.15 (0.61)																			
	Follow-up	4.08 (0.74)																			
**Problem solve with someone to help them address their substance use**					3.97		.02		0.23		0.33		0.10		.23		.07		.77		0.072
	Pre	3.92 (0.86)																			
	Post	4.15 (0.64)																			
	Follow-up	4.25 (0.65)																			
**Composite self-efficacy**					9.28		<.001		0.37		0.38		0.01		.002		.01		>.99		0.15
	Pre	3.66 (0.78)																			
	Post	4.03 (0.78)																			
	Follow-up	4.04 (0.61)																			

^a^Each item begins with “I feel confident in my ability to...”

^b^n=52 for all time points. All self-efficacy items are the same across all survey time points.

^c^ANOVA: analysis of variance.

^d^N/A: not applicable.

**Table 5:** Stigma descriptive statistics and repeated measures ANOVA results.

Stigma		Responses, mean (SD)^a^	Repeated measures ANOVA^b^, F value		*P* value	Post hoc tests, mean difference		Partial eta^2^ for F
**Most people think less of a person who has been in treatment for substance use**				0.22	.80		N/A^c^	0.004
	Pre	3.58 (1.00)						
	Post	3.56 (0.90)						
	Follow-up	3.84 (1.08)						
**I think less of a person who has been in treatment for substance use**				0.03	.96		N/A	0.001
	Pre	1.98 (0.98)						
	Post	1.94 (0.98)						
	Follow-up	1.96 (1.12)						

^a^n=52 for all time points.

^b^ANOVA: analysis of variance.

^c^N/A: not applicable.

Level 3 self-reported behavior results (see Table 6) show no significant change from the presurvey to the follow-up survey in the number of participants that started a conversation with someone they were concerned about regarding their substance use, problem solved with someone to help them address their substance use, and consulted with a health professional about substance use. The lack of significant change led us to examining the responses of the two open-ended questions originally designed to help participants accommodate skill acquisition into the learning experience.

**Table 6:** Self-reported behavior descriptive statistics and repeated measures t test results.

Behavior	Pre, mean (SD)	Follow-up, mean (SD)	Paired sample *t* test (*df*)	*P* value
Started a conversation about substance use with someone you are concerned about	1.46 (2.49)	0.88 (1.94)	1.47 (51)	.15
Problem solved with someone to help them address their substance use	1.37 (2.47)	0.98 (1.92)	1.02 (51)	.31
Consulted with a health professional about substance use	0.92 (2.57)	0.50 (1.38)	1.18 (51)	.24

#### Qualitative Measures

The open-ended question included in the postsurvey was, “Now that you have completed the simulation, please describe a situation that you would have managed differently. What happened and what would you have done differently?” Answers were divided into two parts that included (1) describe a situation and what happened, and (2) how would you have managed it differently?

The open-ended question included in the 6-week follow-up survey was, “Now that you have completed the simulation, can you recall a situation where you used the skills learned in the simulation?” Thematic categories and exemplary statements for the postsurvey and 6-week follow-up survey responses can be found in Tables 7-9. The themes and their relative frequencies that emerged from the coding process previously described are listed in Multimedia Appendix 1.

**Table 7:** Postsurvey responses for describing a situation and what happened (N=80).

Thematic categories			Exemplary statements		Sample size^a^, n (%)
**Presimulation conversation tactics**					
	Approached person in a condescending or attacking manner	“I have tried to approach one of my friend's about their mental health before and I came off too strong and she got offended. Now I feel like I know how to sound more like I'm listening and not make her angry.”		13 (16.3)	
	Choose not to address the person’s substance use	“My roommate in college was beginning to use alcohol as a crutch. Rather than address it, I just let it happen. That person is fine today, but I feel I could have improved their lived experience if I had started a conversation. As the simulation shows, it doesn't take much to start someone thinking about their behavior.”		5 (6.3)	
	Too scared/unsure how to initiate conversation	“The hardest thing is to initiate the conversation. I'm not sure I, someone who is conflict averse, will be able to...”		3 (3.8)	
**No example**					
	Do not have an example	“I have never been in a situation like that, but it definitely gave me some tools I can use if I need to have a similar conversation in the future.”		9 (11.3)	
**Other**					
	Other	N/A^b^		10 (12.5)	

^a^Not all respondents (N=80) clearly answered both parts, hence the smaller number of responses.

^b^N/A: not applicable.

**Table 8:** Postsurvey responses for describing a situation that you would have managed differently (N=80).

Thematic categories and subcategories			Exemplary statements		Sample size^a^, n (%)
**General mention of skills**					
	General mention of using the strategies learned to initiate and guide the conversation in a productive direction	“When confronting a niece about her substance abuse the conversation took a wrong turn and became adversarial so this simulations showed how to approach that differently.”		15 (18.8)	
**Provided general support**					
	Initiated a conversation about substance use	“I would have told my friend to stop drinking so much when he was underage. He could've been saved from a underage drinking ticket.”		12 (15)	
	Offering empathy	“I had a conversation about drug abuse with a family member recently, but instead of empathizing with him, i just attacked him for how his choices were affecting the rest of the family. If I could go back, I'd definitely try to empathize a lot more”		9 (11.3)	
	Provided support	“I would have approached addicts with more of an understanding and hopeful attitude, rather than with pity and reprimand”		3 (3.8)	
	Offered sympathy	“I would have approached addicts with more of an understanding and hopeful attitude, rather than with pity and reprimand”		3 (3.8)	
**Conversational tactics**					
	Focus more on problem solving	“I would have focused more on the problem solving part. I think I tend to go into therapist mode and want to talk allllllll about whats causing it and totally skip over the “now what to we do” until there isn't a lot off time, or its late or whatever. So then the problem solving is a second thought. i would also have eased into it a little more. I confronted someone about it rather bruskly and i think it just started the whole ordeal off wrong...”		9 (11.3)	
	Offer less advice/opinion	“I will definitely be more empathetic with active listening and avoid reaching conclusions and solutions and advice for the situation. It's better to be the guider of conversation to the solution.”		8 (10)	
	Asked more questions	“i would empathize more with my friend instead of giving her advice that pushed her away, and I would ask her questions to help her figure it out by herself.”		4 (5)	
	Approached the person without accusing them	“I myself am a recovering alcoholic. I talk to people all the time about my addiction and frequently have conversations with other people about theirs. I thought it was awesome that even though I've been sober for 9 years, I still learned something from this simulation - if you jump in with accusations (even if they are based on fact or observation) the other party may get defensive and close up.”		4 (5)	
	Discussed consequences of substance use	“approaching the individual without drinking the issues at hand. maybe have the environment different, more in a his comfort zone. Making more of a point of the issues at hand and making it very clear that there is a problem and the consequences at hand. Knowing what words to say and how to approach the individual.”		3 (3.8)	
	Discussed cause of the substance use	“I would have brought up my health concerns for my roommate who recently had picked up smoking as well as figuring out what could possibly be the trigger (stress). Then problem-solving some other ways to deal with it if she agreed it was a problem.”		2 (2.5)	
**Self-reflection**					
	Would have managed my own substance use differently	“I've struggled with addiction problems myself, so looking back, realizing how young I started using would've changed, being aware of my family history, and realizing there was a deeper reason for why I felt the need to alter my state of mind.”		3 (3.8)	
**Other**					
	Other	N/A^b^		6 (7.5)	

^a^Not all respondents (N=80) clearly answered both parts, hence the smaller number of responses.

^b^N/A: not applicable.

**Table 9:** Six-week follow-up survey responses for recalling a situation where you used the skills learned in the simulation and what happened (n=57).

Thematic categories and subcategories			Exemplary statements		Sample size^a^, n (%)
**Did not use skills yet**					
	Have not used skills from the simulation yet	“I have not used the skills in the simulation yet, but I do believe they would be useful if I needed to talk to someone about substance abuse.”		23 (40.4)	
	Mentioned using the simulation but did not give specifics of how or the situation	“I work in HR and while I don’t address substance use specifically, I often mediate and this skills have been useful in helping people find solutions that work for them.”		7 (12.3)	
**General support**					
	Initiated a conversation about substance use	“I felt a little more confident in my skills to do this with a friend I know who has been struggling. After doing the simulation, I had some ideas about how to subtly bring it up without sounding like I was accusing them of anything. I'm not sure if it will result in anything positive but I think our talk helped at least a little”		10 (17.5)	
	Provided a listening ear and support	“An old friend from high school struggled with drug addiction throughout his early college years. It was always swept under the rug until he broke and finally entered rehab and began exercising addiction programs. We talk regularly and always talk about our substance use, we confront each other when we know were on a slippery slope, and have an open dialogue about substance use without judgement.”		5 (8.8)	
**Acted in nonjudgmental way**					
	Adopted a nonjudgmental lens when having a conversation	“I was talking about cannabis use with a friend, and I used what I learned in the simulation. I tried to stay factual with my approach and help problem solve and get into a healthier state. I was non judge-mental and was encouraging and shared my own success with them.”		3 (5.3)	
	Approached the person without accusing them	“I felt a little more confident in my skills to do this with a friend I know who has been struggling. After doing the simulation, I had some ideas about how to subtly bring it up without sounding like I was accusing them of anything. I'm not sure if it will result in anything positive but I think our talk helped at least a little”		2 (3.5)	
**General conversational tactics**					
	Mentioned facts related to substance use	“I talked to a ex friend who's son killed someone because his was drunk driving. The kid is 17 years old. I explained that there were plenty of signs that he had a problem. Plus his living situation did not help..dad has a drinking problem and was addicted to pills. Dad's girlfriend is an addict and dealer. I tried to explain how his home life had an effect on his actions.”		3 (5.3)	
	Discussed consequences of substance use	“I spoke to a friend about their drinking habits. Brought it up as a question, talking about how expensive alcohol is and how I could save a lot of money if I stopped, and about the health benefits of drinking less. I then went on to ask my friend what he thought”		2 (3.5)	
	Told person to slowdown	“My old roommate drank too much and didn't spend time with us, so we told him he should think of slowing down.”		2 (3.5)	
**Instrumental or specific conversational tactics**					
	Talked to person about getting professional help	“My sister was high on drugs and I talked her into going to rehab”		4 (7.0)	
	Offered options or ways to help solve the substance use problem	“Recently I went to a concert and my friends were doing inappropriate drugs. I calmly turned down the offers to partake and used problem solving skills learned in the situation to get my friends to chill out”		3 (5.3)	
	Helped person get out of the bad situation that was fueling substance use	“I talked with a friend about his drinking and helped him to decide to end his relationship with the person enabling him and to move on.”		2 (3.5)	
**Behavioral tactics**					
	Convinced someone to stop/plan to stop substance use	“I approached my girlfriend about cocaine usage and we agreed upon tapering, quitting, and general therapy. I as careful not to be irritating and I feel the varying convos were successful.”		4 (7.0)	

^a^Not all respondents (n=57) clearly answered both parts, hence the smaller number of responses.

## Discussion

### Principal Results

Findings show that participants highly rated the simulation, would recommend it to others, and felt that it was based on relevant scenarios. Participants also reported significant increases in their preparedness and self-efficacy from baseline to follow-up to start a conversation with someone they were concerned about regarding their substance use, avoid upsetting someone while bringing up concerns, focus on observable facts, and problem solve. The results showed a significant overall effect for composite preparedness, likelihood, and self-efficacy. For preparedness, post hoc tests revealed that there was a significant increase in preparedness across all time points (ie, follow up was significantly higher than post and pre, and the post was significantly higher than the pre). For likelihood, although the overall F statistic was significant, there were no individual significant differences between time points due to the adjustment in type I error with the Bonferroni correction. However, the trend was such that the scores increased over time. With self-efficacy, there was also an increase in means over time, but the post and follow-up means were not significantly different than each other, although both were significantly higher than the pretest means.

There were no significant changes in self-reported behaviors regarding starting conversations about substance use with others, problem solving with someone to address their substance use, or consulting with a health professional about substance use. However, the qualitative data provided a more nuanced perspective of the positive impact the simulation had on how people would have applied the skills they learned if they could redo past experiences and how they actually used what they learned as a direct result of the simulation. Perhaps self-reported behaviors were influenced by a number of participants revealing that they themselves were personally dealing with, or had dealt with, substance use issues, which might have incentivized them to enroll in the study as opposed to being specifically concerned about others. Another factor could be that the short 6 week follow-up was not an adequate amount of time to capture changes in behavior. Both of these factors, past exposure to substance use and the short 6-week follow-up period, could have also influenced the small change in personal and perceived public stigma. Despite seeing a slight decrease in negative personal and perceived public stigma toward those experiencing substance use struggles, there was no systematic difference in these measures before and after participating in the simulation.

To extrapolate on the qualitative data, we hypothesize that we would have observed higher use of strategies had we implemented the pilot in a more targeted manner by identifying and recruiting individuals that were specifically interested in this content due to some personal or professional relevancy. In theory, a study sample that expresses a particular need for this type of intervention would lead to a higher number of participants indicating that they used the skills learned in the simulation. This more targeted recruitment would also influence the behavioral results, likely increasing reports of identification, approach, and referral. However, this pilot was conducted without specifically targeting an at-risk group, and we did not screen the participants for content relevancy to their current personal or professional lives prior to recruitment, as this initiative was a part of a general Colorado public health campaign.

These types of virtual role play training simulations have shown to be efficacious in training targeted populations such as health care providers including social workers, educators, school counseling professionals, and students in the health care field [29-34]. Data from these studies show significant improvements in attitudinal constructs related to interpersonal skills acquisition and behavioral changes. Thus, the results in this study, where users were not identified as part of a population of people that were concerned about or affected by family members, friends, or colleagues take on added meaning for they still reported significant changes in attitudes and positive qualitative responses.

### Limitations

There are several limitations of this pilot study; the first being that it is not a randomized controlled trial (RCT). In addition, participants self-selected into the study, which is a common selection bias often found in similar studies. Another limitation is that changes in behaviors were self-reported; therefore, they may not be an accurate indication of the number of conversations participants engaged in about substance use, the number of people they problem solved with, or the number of times they consulted with a health professional. Future studies should use an RCT design and be more rigorous in controlling for possible confounders in participant selection. To confirm this study’s findings and perhaps better observe changes in stigma and help-seeking behavior, future studies should recruit larger sample sizes and should follow up with participants beyond the 6-week timeframe used in this pilot study.

Although this study provides a baseline understanding of the ways in which the simulation may affect changes in preparedness and confidence around initiating conversations about substance use, the results are also limited by the fact that many participants were not presented with opportunities to practice their newly learned skills within the 6-week study timeframe. A similar study with a more targeted audience may have provided better insights in to different aspects of behavior change as a result of the simulation. Future studies should use alternative methods of recruitment targeted at individuals who are more likely to have conversations about substance use arise in their daily life. For example, targeted outreach to support groups for friends and family of individuals who use substances may result in a sample that is more likely to initiate conversations within a 6-week period. In addition, since this study was carried out, Peer Assistance Services, Inc and Kognito Solutions, LLC have developed a third simulation that centers around discussions between adults and adolescents. This simulation could be piloted with parents, coaches, teachers, or other trusted adults who may be in a position to initiate conversations about substance use with young people before it even begins.

There is also one limitation to the One Degree: Shift the Influence application itself that is worth noting. In real life scenarios, not all individuals will be ready or willing to participate in conversations about substance use if approached by a peer, family member, or colleague. Although the virtual humans do provide negative feedback throughout the role play to express when they are unhappy with a chosen conversational technique, these simulations do not address alternative techniques to use if the person approached about their substance use is unwilling to participate in a conversation in the first place. However, simply raising the topic of substance use with someone who is struggling with addiction, regardless of whether or not they are ready to have the conversation, does increase the likelihood of future behavior change [52].

### Conclusions

The preliminary results of this study indicate that, in general, public awareness initiatives may benefit from integrating easy to access, experiential, online virtual human role play simulations. These simulations allow individuals to gain skills and confidence around initiating and managing conversations about substance use by using evidence-based communication strategies such as motivational interviewing. Walker et al [53] points out that, in these critical times of COVID-19, MI is an ideal framework to address substance use. Even before this crisis occurred, alcohol use was the third leading cause of preventable death, and data has shown that the isolation and anxiety around the COVID-19 pandemic have increased the use of alcohol and other substances. Normalizing discussions about substance use is an important first step toward identifying and mitigating risky use. Simulations like *One Degree: Shift the Influence* provide a unique and engaging mode of education aimed at improving individuals’ preparedness and confidence to manage difficult conversations with people that they care about, supplementing existing public awareness initiatives.

Peer Assistance Services, Inc and Kognito Solutions, LLC have also developed online training geared toward health care providers. These training simulations help providers build the skills necessary to integrate an early intervention practice called Screening, Brief Intervention and Referral to Treatment (SBIRT) into their workflow. The brief intervention component of SBIRT requires health care providers to initiate conversations about substance use. Brief interventions are tailored to the patient’s unique needs and concerns, and are rooted in MI techniques. Concurrent efforts to increase and improve the quality of conversations about substance use both in the home and within primary care will lead to overall improvements in health outcomes, reductions in stigma, and earlier referrals to specialized treatment as needed across Colorado.

The results of this study hold promise that this type of new and innovative learning experience can support public health initiatives to cost-effectively reach large numbers of geographically dispersed communities. Online role play simulations can supplement existing substance use prevention and early intervention work, enabling people to manage conversations with others about substance use and motivating them to decrease substance use and seek treatment as necessary. The need for this type of outreach has been delineated in the public health program literature [54,55] and by the Office of the Surgeon General’s Report on Alcohol, Drugs, and Health [56], which emphasized that the substance use care continuum begins with enhancing health, primary prevention, and early intervention.

## Appendix

Multimedia Appendix 1 Correlation matrix of all study variables.

## Abbreviations

ANOVA: analysis of variance
MI: motivational interviewing
NSDUH: National Survey on Drug Use and Health
RCT: randomized controlled trial
SBIRT: Screening, Brief Intervention and Referral to Treatment
