# Supplementary material for: Innovative Virtual Role Play Simulations for Managing Substance Use Conversations: Pilot Study Results and Relevance During and After COVID-19
Source: JMIR Form Res. 2021 Apr 29;5(4):e27164. doi: 10.2196/27164 (PMC8086785; doi:10.2196/27164)
Supplement: Multimedia Appendix 1 [file formative_v5i4e27164_app1.docx]

**Appendix 1. Correlation matrix of all study variables.**

|  | 1 | 2 | 3 | 4 | 5 | 6 | 7 | 8 | 9 | 10 | 11 | 12 | 13 | 14 | 15 | 16 | 17 | 18 |
| --- | --- | --- | --- | --- | --- | --- | --- | --- | --- | --- | --- | --- | --- | --- | --- | --- | --- | --- |
| 1 -Preparedness (Pre) |  |  |  |  |  |  |  |  |  |  |  |  |  |  |  |  |  |  |
| 2 -Preparedness (Post) | .37^**^ |  |  |  |  |  |  |  |  |  |  |  |  |  |  |  |  |  |
| 3 -Preparedness (Follow Up) | .47^**^ | .63^**^ |  |  |  |  |  |  |  |  |  |  |  |  |  |  |  |  |
| 4 -Likelihood (Pre) | .70^**^ | .29^**^ | .42^**^ |  |  |  |  |  |  |  |  |  |  |  |  |  |  |  |
| 5 -Likelihood (Post) | .38^**^ | .65^**^ | .47^**^ | .44^**^ |  |  |  |  |  |  |  |  |  |  |  |  |  |  |
| 6 -Likelihood (Follow Up) | 0.14 | .43^**^ | .70^**^ | 0.21 | .48^**^ |  |  |  |  |  |  |  |  |  |  |  |  |  |
| 7 -Self-Efficacy (Pre) | .82^**^ | .32^**^ | .45^**^ | .66^**^ | .50^**^ | 0.22 |  |  |  |  |  |  |  |  |  |  |  |  |
| 8 -Self-Efficacy (Post) | .29^**^ | .70^**^ | .52^**^ | .23^*^ | .66^**^ | .49^**^ | .34^**^ |  |  |  |  |  |  |  |  |  |  |  |
| 9 -Self-Efficacy (Follow Up) | 0.08 | .52^**^ | .57^**^ | 0.18 | .57^**^ | .78^**^ | 0.16 | .65^**^ |  |  |  |  |  |  |  |  |  |  |
| 10 -Public Stigma (Pre) | -0.10 | 0.13 | 0.06 | -0.04 | 0.02 | -0.13 | -0.13 | 0.08 | -0.17 |  |  |  |  |  |  |  |  |  |
| 11 -Personal Stigma (Post) | -0.15 | -0.22 | -0.29 | -0.36 | -0.26 | -0.20 | -0.10 | -0.22 | -0.24 | .22^*^ |  |  |  |  |  |  |  |  |
| 12 -Public Stigma (Follow-Up) | 0.18 | 0.05 | 0.01 | .30^*^ | .28^*^ | 0.19 | 0.10 | 0.11 | 0.09 | 0.25 | -0.03 |  |  |  |  |  |  |  |
| 13 -Personal Stigma (Follow-Up) | -0.06 | -0.23 | -0.15 | -0.11 | -0.17 | -0.12 | 0.01 | -0.28 | -0.17 | 0.14 | .30^*^ | .31^*^ |  |  |  |  |  |  |
| 14 -Behavior (started convo) (Pre) | 0.18 | 0.02 | 0.03 | 0.21 | 0.13 | -0.08 | .24^*^ | 0.04 | -0.15 | -0.06 | -0.02 | 0.09 | 0.22 |  |  |  |  |  |
| 15 -Behavior (problem-solved) (Pre) | 0.17 | -0.01 | 0.01 | 0.14 | 0.13 | -0.05 | .23^*^ | 0.04 | -0.17 | -0.13 | 0.01 | 0.06 | .28^*^ | .90^**^ |  |  |  |  |
| 16 -Behavior (consulted health pro) (Pre) | 0.10 | 0.12 | 0.03 | 0.05 | 0.14 | -0.08 | 0.20 | 0.13 | -0.11 | -0.19 | -0.09 | -0.04 | 0.15 | .70^**^ | .79^**^ |  |  |  |
| 17 -Behavior (started convo) (Follow Up) | 0.06 | -0.06 | 0.21 | 0.15 | 0.19 | 0.25 | 0.05 | 0.02 | 0.03 | -0.11 | -0.20 | 0.03 | 0.03 | 0.21 | 0.23 | 0.17 |  |  |
| 18 -Behavior (problem-solved) (Follow Up) | 0.05 | -0.18 | 0.18 | 0.07 | 0.17 | .32^*^ | 0.06 | -0.04 | 0.09 | -0.19 | -0.19 | 0.02 | 0.03 | 0.04 | 0.16 | 0.03 | .84^**^ |  |
| 19 -Behavior (consulted health pro) (Follow Up) | 0.13 | 0.02 | 0.05 | -0.01 | 0.10 | 0.06 | 0.18 | .28^*^ | 0.02 | -0.11 | -0.13 | 0.11 | 0.15 | .29^*^ | .38^**^ | .27^*^ | .33^*^ | 0.22 |
